# Manganese-Induced Neurotoxicity: New Insights Into the Triad of Protein Misfolding, Mitochondrial Impairment, and Neuroinflammation

**DOI:** 10.3389/fnins.2019.00654

**Published:** 2019-06-26

**Authors:** Dilshan S. Harischandra, Shivani Ghaisas, Gary Zenitsky, Huajun Jin, Arthi Kanthasamy, Vellareddy Anantharam, Anumantha G. Kanthasamy

**Affiliations:** Department of Biomedical Sciences, Parkinson’s Disorder Research Laboratory, Iowa State University, Ames, IA, United States

**Keywords:** manganese neurotoxicity, Parkinson’s disease, protein aggregation, exosome, cell-to-cell transmission and neuroinflammation

## Abstract

Occupational or environmental exposure to manganese (Mn) can lead to the development of “Manganism,” a neurological condition showing certain motor symptoms similar to Parkinson’s disease (PD). Like PD, Mn toxicity is seen in the central nervous system mainly affecting nigrostriatal neuronal circuitry and subsequent behavioral and motor impairments. Since the first report of Mn-induced toxicity in 1837, various experimental and epidemiological studies have been conducted to understand this disorder. While early investigations focused on the impact of high concentrations of Mn on the mitochondria and subsequent oxidative stress, current studies have attempted to elucidate the cellular and molecular pathways involved in Mn toxicity. In fact, recent reports suggest the involvement of Mn in the misfolding of proteins such as α-synuclein and amyloid, thus providing credence to the theory that environmental exposure to toxicants can either initiate or propagate neurodegenerative processes by interfering with disease-specific proteins. Besides manganism and PD, Mn has also been implicated in other neurological diseases such as Huntington’s and prion diseases. While many reviews have focused on Mn homeostasis, the aim of this review is to concisely synthesize what we know about its effect primarily on the nervous system with respect to its role in protein misfolding, mitochondrial dysfunction, and consequently, neuroinflammation and neurodegeneration. Based on the current evidence, we propose a ‘Mn Mechanistic Neurotoxic Triad’ comprising (1) mitochondrial dysfunction and oxidative stress, (2) protein trafficking and misfolding, and (3) neuroinflammation.

## Metals in Biology

At least 13 metals have been identified as essential for life, and four of these (sodium, potassium, magnesium, and calcium) occur in large amounts. The remaining nine trace metals (manganese, iron, cobalt, vanadium, chromium, molybdenum, nickel, copper, and zinc) assume vital roles in building organic biomolecules as well as in regulating biological functions. In the last couple of decades, the importance of metal ions in protein biology has been an increasingly attractive research subject given their association with many human diseases, for which metals have been identified as a causative or stimulatory agent. Metals are essential because of their integral role in enzymes that catalyze the basic metabolic or biochemical processes shared by all forms of life on earth. About one-third of all proteins depend on metal ions to carry out their biological functions (Holm et al., 1996). When considering all six classes of enzymes – oxidoreductases, transferases, hydrolases, lyases, isomerases, and ligases – over 40% of all enzymes contain metals (Andreini et al., 2008). Moreover, the chemistry of metals allows for a broader set of protein-metal reactions. For instance, redox-active metal ions are often interchangeable depending on the metal concentration and their affinities to protein. Protein affinities for trace metals are substantially determined by universal series, which for divalent metals is the Irving-Williams series (Mn^2+^ < Fe^2+^ < Co^2+^ < Ni^2+^ < Cu^2+^ > Zn^2+^), wherein Cu^2+^ is highly competitive and can replace lower order metals (Tottey et al., 2008).

These “metalloproteins” are involved in many key biological processes, such as gas transport, cell respiration, antioxidant defense, photosynthesis, and many other vital redox reactions driven by their interaction with metals. Well-characterized examples for redox-active metalloprotein systems are blue-copper proteins, heme-binding proteins and iron-sulfur-cluster proteins. Moreover, recent advances in synthetic chemistry have focused on the study of metal sites in metalloproteins and metalloenzymes to influence biological processes in the battle against many daunting human diseases. Advanced medicinal chemistry approaches have given us new, innovative medicinal applications of metal complexes and organometallic agents. Prime examples for such uses of metals include platinum-containing anticancer drugs (e.g., Cisplatin), lithium-containing depression drugs (e.g., Camcolit), and manganese (Mn)-containing anticancer drugs (e.g., SOD mimics) (Farrell, 2003).

Presumably, all metalloproteins would bind to their desired metal ligands, and this binding can regulate their folding. However, despite the wealth of structural information, the coupled protein-folding, metal-binding pathways for metalloproteins remain largely unknown (Wittung-Stafshede, 2002). Proper protein folding is critical to the conformational integrity and function of proteins. However, metal ligand binding can also induce undesirable structural transitions in proteins that eventually lead to the formation of pathological protein aggregates. Indeed, the pathologies of Alzheimer’s disease (AD), PD, and prion diseases are linked to abnormal misfolding of otherwise harmless neural proteins. For example, in AD, increased levels of metals, such as Cu^2+^ and Zn^2+^, are linked to the aggregation of Aβ protein *in vitro* (Kenche and Barnham, 2011). The theory of metal-induced aggregation is supported by numerous studies tying metal concentrations in the brain with AD, PD, and amyotrophic lateral sclerosis (ALS) in *in vivo* and *in vitro* studies employing recombinant proteins (Brown et al., 2005; Brown, 2011).

In this review, we will focus on α-synuclein, one of the major proteins implicated in PD, and its interactions with metals, specifically, its interaction with Mn in oxidative stress, protein aggregation and neurodegeneration.

## Parkinson’s Disease

Parkinson’s disease is recognized as the second most prevalent neurodegenerative disorder after AD, affecting roughly 1% of the population over the age of 65. It is also the most common movement disorder in the elderly, resulting in bradykinesia, resting tremor, and rigidity (Lotharius and Brundin, 2002). Several non-motor symptoms involving the autonomic nervous system have also been gaining attention (Pfeiffer, 2009; Schapira et al., 2017). PD is characterized histopathologically by the degeneration of dopaminergic neurons in the substantia nigra pars compacta (SNpc), leading to the progressive loss of the neurotransmitter dopamine and hence the above-mentioned cardinal motor deficits. Even though PD is also often associated with the abnormal accumulation of misfolded proteins, primarily α-synuclein, in cytoplasmic inclusions called Lewy bodies (LB) and Lewy neurites, the pathophysiological association between Lewy pathology and disease pathogenesis is not well understood. Similar neuropathological lesions involving the deposition of abnormal proteins also characterize other neurological disorders (Ross and Poirier, 2004), including AD (Kotzbauer et al., 2001; Uchikado et al., 2006), Lewy body dementia (LBD) (McKeith et al., 2004), Huntington’s disease (HD) (Davis et al., 2014), multiple system atrophy (MSA) (Shoji et al., 2000), and some prion diseases (Aguzzi and Calella, 2009; Aguzzi and O’Connor, 2010).

Although aging remains the greatest risk factor for idiopathic PD, a small fraction of patients were identified with familial PD, which is caused by mutations in several genes associated with protein metabolism, ion transport and mitochondrial function. Genes associated with early-onset PD include *α-synuclein* (PARK1), *parkin* (PARK-2), *PINK1* (PARK6), *DJ-1* (PARK7) and *ATP13A2* (PARK9), while those linked with late-onset PD include *LRRK2* (PARK8) and *VPS35* (PARK-17) (Dawson et al., 2010; Roth, 2014). A growing number of epidemiological and clinical studies have identified environmental risk factors for PD, including repeated head trauma, heavy metal toxicity, pesticide toxicity, obesity, and some surrogate measures such as rural living, contaminated well water, substance abuse, and farming (Priyadarshi et al., 2001; Dick et al., 2007). Interestingly, some of these environmental triggers and toxins induce pathophysiological features that mimic PD when they are administered in experimental animal settings. One such toxin is MPTP (methyl-4-phenyl-1,2,3,6-tetrahydropyridine), a compound produced as an impurity during synthesis of the illicit narcotic desmethylprodine. MPTP causes chronic and severe Parkinsonism by selectively damaging the SN, resulting in PD-related motor deficits (Langston et al., 1983; Ballard et al., 1985; Appendino et al., 2014). Other compounds widely used in experimental models to study the etiopathogenesis of PD include the narcotic methamphetamine, the dopamine derivative 6-hydroxydopamine, and pesticides such as rotenone, paraquat, and dieldrin. These neurotoxins cause nigrostriatal cell death by interfering with mitochondrial function, inducing oxidative stress, protein aggregation, and modifying proteasomal function (Kanthasamy et al., 2008; Latchoumycandane et al., 2011; Ghosh et al., 2013; Jin et al., 2015b). In addition, exposure to heavy metals (e.g., iron, lead, mercury, cadmium, arsenic, and Mn) and metal-based nanoparticles increases the risk of PD through the neurotoxic accumulation of metals in the SNpc and by increasing oxidative stress-induced apoptosis (Afeseh Ngwa et al., 2009; Milatovic et al., 2009; Afeseh Ngwa et al., 2011; Kanthasamy et al., 2012; Aboud et al., 2014; Harischandra et al., 2015a).

## Manganese

Manganese is considered to be a key inhaled environmental pollutant as well as a putative risk factor for environmentally linked PD and related neurodegenerative disorders. Being the 12th most abundant element and composing approximately 0.1% of the earth’s crust, Mn is ubiquitously present in the environment (Martinez-Finley et al., 2012). Besides the earth’s crust, other Mn sources include direct atmospheric deposition, wash-off from plant and other surfaces, leaching from plant tissues, ocean spray, and volcanic activity. Mn occurs in trace amounts in all body tissues as it is essential for many ubiquitous enzymatic reactions, including the synthesis of amino acids (AA), lipids, proteins, and carbohydrates. It also plays a key nutritional role in bone growth, fat and carbohydrate metabolism, blood sugar regulation, and calcium absorption (Bowman et al., 2011). Being present in whole grains, rice, nuts, tea, leafy green vegetables, and Mn-containing nutritional supplements, the primary route of Mn exposure in humans is through dietary intake. The abundance of Mn-enriched food in the typical daily diet makes it relatively easy to accrue the daily reference intake (DRI) of 2.3 mg/day for men and 1.8 mg/day for women (Aschner and Aschner, 2005), thereby minimizing the risk of Mn deficiency-related birth defects, impaired fertility, osteoporosis, and enhanced susceptibility to seizures (Dendle, 2001; Aschner and Aschner, 2005; Sarban et al., 2007).

Despite its nutritional benefits, prenatal and postnatal overexposure to Mn affects infant neurodevelopment, exemplifying its role as both an essential nutrient and a toxicant (Zota et al., 2009; Claus Henn et al., 2010). High Mn exposure in early life is associated with poor cognitive performance, especially in the verbal domain of children (Menezes-Filho et al., 2011). In older cohorts, chronic excessive exposure to occupational or environmental sources of Mn causes manganism, which is characterized by a severe neurological deficit that often resembles the involuntary extrapyramidal symptoms associated with PD (Kwakye et al., 2015). Couper (1837), at the University of Glasgow, reported the first case of Mn-induced neurotoxicity, which was discovered in employees of Charles Tennant and Co., a manufacturer of bleaching powder. Later, public awareness of Mn neurotoxicity arose as more clinical studies identified a PD-like syndrome in workers employed at a Mn ore-crushing plant and a ferromanganese factory (Cook et al., 1974; Huang et al., 1989). In addition, Rodier (1955) detailed clinical features of manganese neurotoxicity in Moroccan miners. Since then, the commercial applications for Mn have broadened considerably so that now Mn exposure also occurs through its use as an additive in gasoline (methylcyclopentadienyl manganese tricarbonyl, MMT) and fertilizers, and as manganese violet in paint and cosmetics (Martinez-Finley et al., 2012). Mn neurotoxicity occurs often in agricultural workers exposed to organic Mn-containing pesticides, such as manganese ethylene-bis-dithiocarbamate (Maneb) and in chronic abusers of the street drug ‘Bazooka’, a cocaine-based drug contaminated with manganese carbonate (Ensing, 1985). The other major anthropogenic sources of environmental Mn include municipal wastewater discharge, welding, mining and mineral processing, metal (alloy, steel, and iron) manufacturing emissions, fossil fuel combustion, and dry-cell manufacturing. Although the precise mechanisms through which Mn is absorbed into the body are not fully understood, it is known to accumulate predominantly in the brain’s basal ganglia region. Beyond the many commonalities shared between manganism and PD, it is also worth pointing out their differences. Behaviorally, manganism is mainly characterized by milder and less frequent resting tremor that tends to be postural or actional, a propensity to fall backward, excessive salivation, and frequent dystonia consisting of facial grimacing, hand dystonia, and/or plantar flexion (Calne et al., 1994). Manganism patients were also reported to have symptoms of irritability, emotional lability, and hallucinations and psychoses referred to as “manganese madness” (Huang, 2007). Pathologically, Mn neurotoxicity affects primarily neurons in both the globus pallidus and striatum, whereas PD predominantly affects dopaminergic neurons in the SNpc (Roth, 2014). Therefore, in fact, the PD-like behavior deficits in manganism result from Mn’s capability to suppress dopamine release from the striatum, thus generating behavioral dysfunctions common to both PD and manganism (Kim et al., 2002; Racette et al., 2005; Fitsanakis et al., 2006; Roth et al., 2013).

## Manganese Homeostasis

The homeostasis of Mn and other metal ions is maintained through tightly regulated mechanisms of uptake, storage, and secretion that strictly limit their abundance in the cellular compartment. The distribution and neurotoxicity of Mn is governed largely by the routes of exposure, which are primarily ingestion and inhalation. In humans, the primary route of exposure is through Mn-enriched food and well water. However, the molecular mechanisms of oral Mn absorption are not well understood. Roughly 3–5% of the Mn ingested gets absorbed into the body from the gastrointestinal tract (GIT) (Finley et al., 1994). Under homeostatic conditions, Mn enters the portal circulation through either passive diffusion (Bell et al., 1989) or active transport via divalent metal transporter 1 (DMT1) (Erikson and Aschner, 2006; Fitsanakis et al., 2007), which was the first mammalian transmembrane iron transporter to be identified. Formerly known as Nramp2 or DCT1, DMT1 is a 12-transmembrane domain protein responsible for the uptake of various divalent metals including Fe^2+^, Mn^2+^, Zn^2+^, Co^2+^, and Ni^2+^, and it transfers iron across the apical surface of intestinal cells and out via transferrin (Tf)-cycle endosomes (Andrews, 1999). Besides using a mechanism similar to that for iron, there are no known metal transporters specific for transporting Mn into cells. In plasma, approximately 80% of Mn^2+^ is bound to α-macroglobulin or albumin, while only a small fraction (<1%) of Mn^3+^ is bound to Tf. It has been proposed that, like iron, Mn in plasma is oxidized from Mn^2+^ to the Mn^3+^ valence state by the ferroxidase enzyme ceruloplasmin and loaded onto plasma Tf for circulating into tissues (Davidsson et al., 1989). Circulating Mn diffuses throughout the body, including bone, kidney, pancreas, liver, and brain (Martinez-Finley et al., 2012).

Once in the brain, Mn^3+^ entry into neurons occurs by the Tf-Mn^3+^ complex binding to the transferring receptor (TfR) and it becomes localized in endosomes. Subsequent recruitment of v-ATPases acidifies endosomes and dissociates Mn^3+^ from the Tf/TfR complex, reducing it to Mn^2+^, which is quite stable at physiological pH, and thereafter, neuronal transport occurs via DMT1 independent of the Tf pathway. In the brain, DMT1 is highly expressed in the DA-rich basal ganglia, putamen, cortex, and SN (Huang et al., 2004; Salazar et al., 2008), which may account for Mn’s pattern of accumulation and neurotoxicity. Other primary transport mechanisms for Mn are through capillary endothelial cells of the blood-brain-barrier (BBB) (Crossgrove et al., 2003) or through the CSF via the choroid plexus (Murphy et al., 1991). Since Mn neurotoxicity primarily occurs through occupational exposure, such as inhalation of Mn fumes or dust in welding, dry-cell battery manufacturing, and the smelting industry, the nasal passage through the olfactory epithelium to the olfactory nerve is another major Mn transport mechanism into the brain (Tjalve et al., 1996). In fact, DMT1 is highly expressed in the olfactory epithelium and is required for Mn transport across the olfactory epithelium, as has been shown in the rat (Thompson et al., 2007). Evidence also exists for Mn transport into the central nervous system (CNS) through store-operated calcium channels (Crossgrove and Yokel, 2005), ionotropic glutamate receptor calcium channels (Kannurpatti et al., 2000), and Mn citrate transporters (Crossgrove et al., 2003).

Another mechanism regulating Mn homeostasis in the brain involves Mn being transferred with high affinity into cells by the Zinc transporters ZIP-8 and ZIP-14, which are Zrt-/Irt-related protein (ZIP) family metal transporters encoded by SLC39A8 and SLC39A14, respectively. These transporters are highly expressed in the liver, duodenum, kidney, and testis, and are localized on apical surfaces of brain capillaries (Girijashanker et al., 2008; Wang et al., 2012). Taking advantage of its particular magnetic properties, Aoki et al. (2004) employed magnetic resonance imaging (MRI) to show that Mn uptake also occurs through the choroid plexus. One day after they systemically administered Mn^2^^+^ to rats, the distribution of Mn in the brain extended to the olfactory bulb, cortex, basal forebrain, and basal ganglia, overlapping specific brain structures vulnerable to Mn-induced neurotoxicity (Aoki et al., 2004). In cells such as neurons and astrocytes, toxic accumulations of Mn are found primarily in the mitochondria, heterochromatin, and nucleoli (Lai et al., 1999; Morello et al., 2008).

Mn also shares the Ca^2+^ uniporter mechanism and the rapid mode (RaM) of Ca^2+^ uptake of mitochondrial calcium influx, resulting in Mn sequestration in mitochondria, which gets removed only very slowly from the brain (Gavin et al., 1990). This Mn accumulation inhibits the efflux of calcium, decreases MAO activity, and inhibits the respiratory chain and ATP production (Zhang et al., 2003), which may partly explain the role of mitochondrial dysfunction in Mn neurotoxicity. Previously, Mn detoxification and efflux from cells was thought to be primarily regulated by ferroportin (Fpn), also known as HFE4, MTP1, and IREG1, which are proteins encoded by the SLC40A1 gene. Although Fpn was initially identified as the iron exporter, more recent findings suggest that Fpn also interacts with Mn, zinc, and cobalt to export them from the cell (Troadec et al., 2010; Yin et al., 2010; Madejczyk and Ballatori, 2012). Furthermore, Mn exposure increases Fpn mRNA levels in mouse bone marrow macrophages (Troadec et al., 2010) and it significantly increases Fpn protein levels in HEK293T cells (Yin et al., 2010). Increasing Fpn levels were linked to reduced Mn accumulation in both the cerebellum and cortex of mice treated with Mn (Yin et al., 2010), further confirming that Fpn removes Mn and reduces Mn-induced neurotoxicity.

Recently, the secretory pathway of the Ca^2+^/Mn^2+^ ATPases SPCA1 and SPCA2, which are localized at the Golgi, was suggested as an alternative way of cytosolic Mn detoxification by sequestering into the Golgi lumen (Sepulveda et al., 2009). Overexpressing SPCA1 in HEK293T cells conferred tolerance of manganese (Mn^2+^) toxicity by facilitating Mn^2+^ accumulation in the Golgi, thereby increasing cell viability (Leitch et al., 2011). However, the degree to which SPCA1 and SPCA2 regulate Mn homeostasis has yet to be determined. Another mode for Mn egress through Golgi has been attributed to SLC30A10 in humans (Tuschl et al., 2012). Recently, SLC30A10 was shown to be localized on the cell surface where it acted as a Mn efflux transporter to reduce cellular Mn levels and protect against Mn-induced toxicity (Leyva-Illades et al., 2014). Mutations in the SLC30A10 gene have been associated with hepatic cirrhosis, dystonia, polycythemia, Parkinsonian-like gait disturbances, and hypermanganesemia in cases unrelated to environmental Mn exposure (Tuschl et al., 2012). A genome-wide association study mapping genes involved in regulating Mn homeostasis mapped serum Mn levels to SLC30A10 (Ng et al., 2015). Along with its expression in the liver and CNS, SLC30A10 is also expressed in the GIT. Interestingly, this transporter is present mainly on the apical surface of enterocytes that line the GIT and presumably help transport Mn to the lumen. In fact, it is the liver and GIT that are primarily responsible for maintaining Mn homeostasis in the body as indicated by whole body as well as endoderm-specific SLC30A10 knockouts (KOs) resulting in hypermanganesemia, while pan neuronal/glial SLC30A10 KOs produce normal levels of Mn in the CNS (Taylor et al., 2019). The authors also found that a lack of SLC30A10 in the CNS led to an increased accumulation of Mn in the basal ganglia and thalamus when these mice were exposed to elevated Mn levels. Importantly, these recent discoveries involving SLC30A10 and its mutations reinforce its crucial role in humans as a Mn transporter, broadening our understanding of familial Parkinsonism as a result of *SLC30A10* mutations.

**FIGURE 1:**
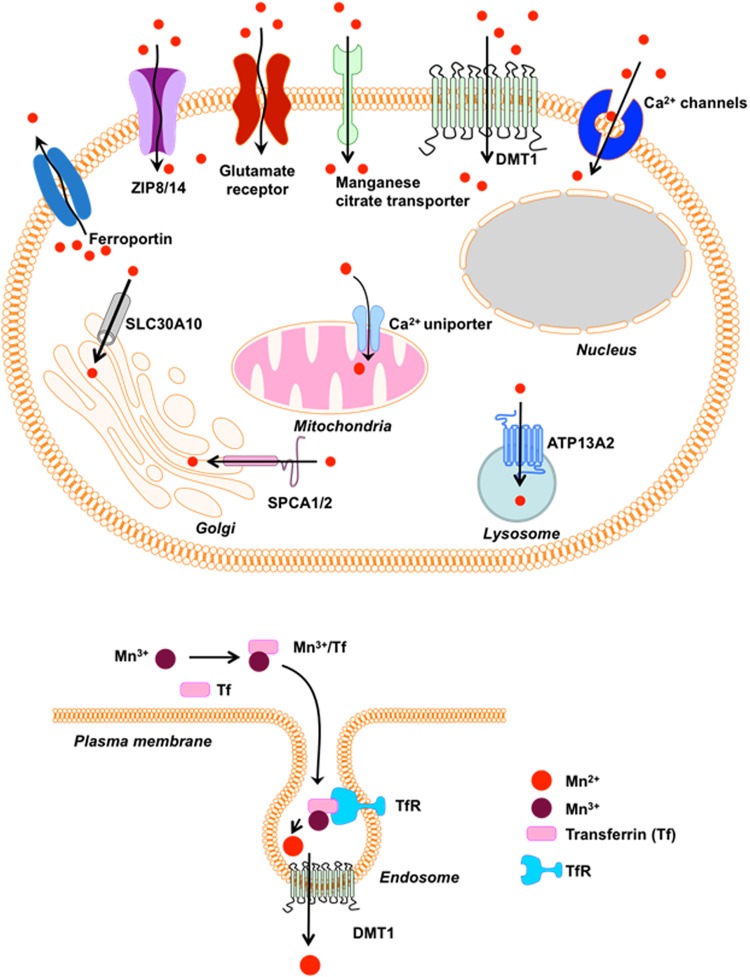
Receptors/Channels involved in Mn homeostasis. Various cellular receptors such as divalent metal transporter 1 (DMT1) and transferrin receptor (TfR), as well as ion channels, store-operated Ca^+2^ channels (SOCC) or voltage-gated Ca^+2^ channels (VGCC) that facilitate the entry of divalent Mn into cells, whereas SLC40A (ferroportin) and Ca^+2^ facilitate its expulsion from cells and mitochondria, respectively. Mn^+2^ is passively transported via VGCC and glutamate-activated ion channels while Mn^+3^ entry is facilitated via transferrin.

The p-type transmembrane ATPase protein ATP13A2 (or PARK9) located at the lysosome also protects cells from Mn-induced toxicity (Tan et al., 2011). Although ATP13A2’s function in mammalian cells remains elusive, loss-of-function mutations in ATP13A2 cause Kufor-Rakeb Syndrome (KRS), an autosomal recessive form of early-onset Parkinsonism with pyramidal degeneration and dementia (Ramirez et al., 2006). Overexpression of wild-type (WT) ATP13A2, but not KRS pathogenic ATP13A2 mutants, protects mammalian cell lines and primary rat neuronal cultures from Mn^2+^-induced cell death by reducing intracellular Mn concentrations and cytochrome c release, suggesting a role of ATP13A2 in Mn detoxification and homeostasis (Tan et al., 2011). A summary of the above-mentioned receptors and channels involved in cellular Mn homeostasis appears in Figure 1.

## Manganese and α-Synuclein Protein Misfolding

Belonging to a family that includes β- and γ-synuclein, α-synuclein (αSyn) is a small 140-AA, highly conserved vertebrate protein encoded by a single 7-exon gene located on chromosome 4. It is predominantly a neuronal protein expressed in presynaptic terminals throughout the mammalian brain and CSF where it is estimated to account for as much as 1% of total protein in soluble cytosolic brain fractions. Functionally, αSyn remains poorly understood, but emerging evidence points to roles in membrane trafficking, dopamine regulation, and synaptic plasticity. The link between αSyn and PD pathogenesis is based on case studies of familial and sporadic PD patients presenting with misfolded αSyn-rich Lewy pathology during autopsy (Poulopoulos et al., 2012). Also, compelling evidence demonstrates that mutations in the gene encoding αSyn are directly linked to the onset of PD (Liu et al., 2012). Furthermore, rare familial forms of PD also have been linked to the overexpression of αSyn due to *SNCA* gene duplication and triplication.

The aggregation and fibrillation of αSyn, forming intracellular proteinaceous aggregates, have been implicated in several other neurodegenerative disorders besides PD, including LBD, Lewy body variant of AD, MSA, and Hallervorden–Spatz disease. The idea that extracellular αSyn species can accelerate the spread of PD pathology throughout the brain gained much consideration with the findings of host-to-graft propagation of αSyn-positive Lewy pathology in fetal ventral mesencephalic and embryonic nigral neurons transplanted in human PD patients (Kordower et al., 2008; Li et al., 2008) and misfolded αSyn species in human CSF and plasma (El-Agnaf et al., 2003; Kordower et al., 2008). Although multiple studies have hypothesized the intercellular transmission of pathological αSyn species in PD (Lee et al., 2008; Desplats et al., 2009; Dunning et al., 2013), its exact mechanistic role in disease pathogenesis and related synucleinopathies largely remains unknown. Available *in vitro* evidence thus far postulates that extracellular αSyn induces pathogenic actions by multiple mechanisms including, but not limited to, the triggering of neuroinflammatory responses and mitochondrial dysfunction leading to neurodegenerative processes (Su et al., 2008; Emmanouilidou et al., 2010).

**FIGURE 2:**
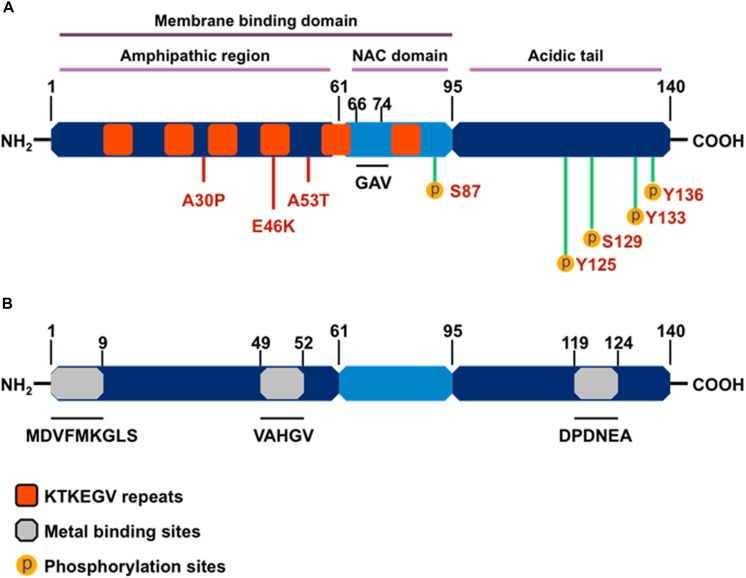
The structure of α-synuclein. **(A)** Orange boxes denote the characteristic KTKEGV repeat and red lines denote amino acid mutations seen in familial PD patients. The central hydrophobic core (middle light blue), or NAC domain, promotes α-synuclein aggregation. The C-terminal domain (right dark blue) is the acidic tail and contains most known phosphorylation sites. **(B)** Gray boxes denote known metal-binding sites in the α-synuclein structure.

As a member of the family of intrinsically unstructured proteins, αSyn is natively unfolded and lacks a defined secondary protein structure. However, upon interaction with lipid membranes, it adopts an α-helical conformational change, and under conditions that trigger aggregation, αSyn undertakes the characteristic crossed β-conformation and self-aggregates into soluble oligomers, which gradually form insoluble amyloid-like fibrils. The αSyn protein comprises three main structural domains (Figure 2): (1) an N-terminal amphipathic region, (2) an amyloid-binding central domain (NAC), and (3) a C-terminal acidic tail. The N-terminus (residues 1-60) contains four series of 11-AA repeats containing the highly conserved consensus sequence KTKEGV, which also is important for α-helix conformation upon binding to phospholipid membranes. The core NAC region (residues 61–91) is important in protein aggregation and it also contains two additional KTKEGV repeats. Within the NAC, a hydrophobic GAV peptide motif (residues 66–74), consisting of Ala, Val, and Gly AA residues, has been identified as the required core for human αSyn protein fibrillization and cytotoxicity (Du et al., 2006). Finally, the proline-rich C-terminus (residues 91–140) is highly acidic and accounts for the intrinsically disordered properties of αSyn (Harischandra et al., 2015a). The N-terminal and NAC regions form αSyn’s membrane binding domain, whereas the C-terminal region is believed to contain protein–protein and protein–small molecule interaction sites.

Importantly, αSyn wields its metalloprotein properties through its three metal-binding sites: two each at the N-terminus and one at the C-terminus. A systematic analysis of mono-, di-, and trivalent metal ligands (Li^+^, K^+^, Na^+^, Cs^+^, Ca^2+^, Co^2+^, Cd^2+^, Cu^2+^, Fe^2+^, Mg^2+^, Mn^2+^, Zn^2+^, Co^3+^, Al^3+^, and Fe^3+^) revealed that metal binding induces conformational changes that cause normally benign αSyn protein to aggregate (Uversky et al., 2001). Of the 15 metal cations studied, Uversky et al. (2001) determined Al^3+^ to be the most effective stimulator of protein fibril formation followed by Cu^2+^, Fe^2+^, Co^3+^, and Mn^2+^, with each causing conformational changes detectable by intrinsic protein fluorescence and far UV-circular dichroism. Furthermore, Uversky’s team also showed that Mn^3+^ induced immediate di-tyrosine formation, suggesting that Mn is responsible for the metal-induced oxidation of αSyn. Among the three metal-binding sites, those located at the N-terminus, specifically the ^1^MDVFMKGLS^9^ and ^48^VAHGV^52^ regions, demonstrated high-affinity binding for Cu^2+^ (*K*_d_ ∼ 0.1 μM) (Rasia et al., 2005), whereas metal-interaction sites near residues 49–52 and residues 110–140 are known to bind with divalent metals like Mn (Uversky et al., 2001; Binolfi et al., 2006, 2008). In a detailed study, the metal ions Mn^2+^, Fe^2+^, Co^2+^, and Ni^2+^ bound preferentially to the ^119^DPDNEA^124^ motif, in which Asp121 acted as the main anchoring site with low affinity (mM) to metal ligands (Binolfi et al., 2006). These discoveries on the structural components of αSyn’s interaction with metals strengthen the link between metal neurotoxicity and PD, further suggesting that metal dyshomeostasis plays an even more important role in the development of neurodegenerative disorders than previously acknowledged (Binolfi et al., 2006).

**FIGURE 3:**
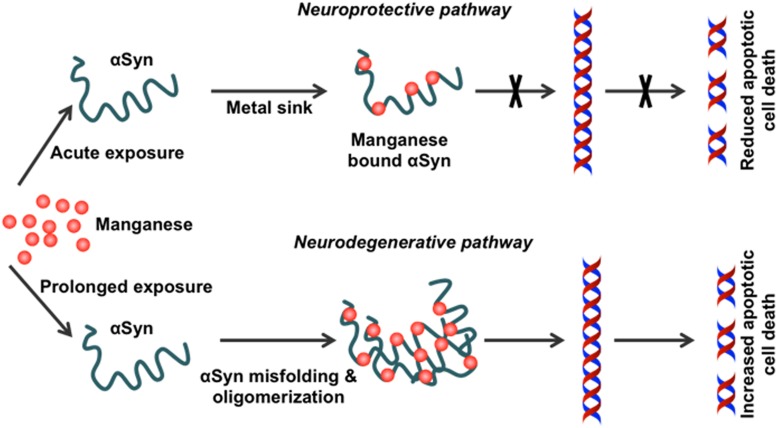
Effect of chronic Mn overexposure on α-synuclein misfolding. Metal-binding sites on α-synuclein (αSyn) allow it to become a metal sink for free-roaming metals in cells. During an acute exposure to Mn, free-roaming Mn binds to the metal-binding sites on the C-terminus of this protein. In this way, αSyn effectively works as a chelator for different metal ions, including Mn. However, continued exposure to Mn can saturate this chelating property. Once saturation occurs following additional binding of Mn to the C-terminus, the natively conformed protein misfolds. Misfolded αSyn leads to the production of pro-inflammatory factors. Thus, Mn overexposure leads to progressive protein misfolding in the neurons and induces inflammation and finally neurodegeneration.

Our *in vitro* studies show that physiological levels of human WT αSyn attenuate acute Mn-induced dopaminergic neuronal degeneration. However, this neuroprotective effect is diminished by chronic exposure to Mn toxicity, which accelerates αSyn misfolding (Harischandra et al., 2015a). Furthermore, using a genetically modified *Caenorhabditis elegans* model system, Bornhorst et al. (2014) reported enhanced Mn accumulation and oxidative stress in pdr1 and djr1.1 mutants, which were reduced by αSyn expression. This protective role of αSyn in Mn-induced neurotoxicity was further validated using αSyn transgenic animals (Yan et al., 2019). By treating αSyn KO (αSyn^–/–^) and WT (αSyn^+/+^) mice with different Mn concentrations, this study demonstrated that the presence of αSyn ameliorates high-dose Mn-induced neurotoxicity. Taken together, these findings point to a novel, neuroprotective role of WT αSyn in attenuating acute Mn toxicity, an effect which may stem directly from its metal-binding capability (Figure 3).

Although the physiological role of αSyn with respect to Mn toxicity still needs to be fully validated, the effects of Mn on αSyn expression, aggregation, and subsequent cytotoxicity have been studied in *in vitro*, *in vivo*, and *ex vivo* models of PD (Gitler et al., 2009; Verina et al., 2013; Xu et al., 2013). Studies conducted with neuronal cell culture models show that Mn treatment upregulates cellular αSyn levels and leads to αSyn aggregation (Cai et al., 2010). In contrast, knocking down αSyn using antisense αSyn treatment (Li et al., 2010) or siRNA (Cai et al., 2010) can reverse Mn-induced cytotoxicity. In parallel studies, overexpressing αSyn in rat mesencephalic cells (MES 23.5) not only enhanced their susceptibility to Mn exposure (Prabhakaran et al., 2011), but also attenuated Mn release from Mn-treated cells without significantly attenuating the major Mn transporter proteins DMT1, VGCC, and Fpn1 (Ducic et al., 2015). Thus, these studies further suggest that αSyn’s metal-binding capacity serves as an intracellular Mn store that helps to regulate free-roaming Mn cations.

## Manganese and Endosomal Trafficking

Accumulating evidence indicates that secretion and cell-to-cell trafficking of pathological forms of αSyn may explain the typical progression of PD. In particular, vesicular trafficking has attracted considerable attention as an initiator or enhancer of the neurodegenerative process underlying PD. Dysfunction of the cellular trafficking pathway can compromise synaptic function and lead to the accumulation of misfolded αSyn. Similarly, changes in endosomal sorting and degradation greatly influence the intracellular trafficking of misfolded proteins, thereby enabling the cell-to-cell transmission of toxic αSyn species in a prion-like manner. Recent genetic studies also suggest that defects of endolysosomal function could disrupt αSyn homeostasis and mitochondrial function, causing neurotoxicity through unknown mechanisms (Kett and Dauer, 2016). Indeed, several PD-linked gene mutations or polymorphisms (DNAJC13/RME-8, VPS35, ATP13A2, ATP6AP2, RAB7L1, GBA, GAK, LRRK2) interrupt protein trafficking and degradation via the endosomal pathway (Perrett et al., 2015), highlighting the importance of the endosomal pathway in the progression of neurodegenerative disease.

It has been shown that αSyn overexpression blocks endoplasmic reticulum (ER)-to-Golgi vesicular trafficking (Cooper et al., 2006) and that αSyn is functionally associated with endocytic vesicular trafficking, retromer complex proteins, phosphatases, and Rab GTPases (Breda et al., 2015; Chung et al., 2017). In this regard, recent attempts to identify molecular regulators of αSyn oligomerization have identified several Rab proteins, including Rab8b, Rab11a, Rab13, Slp5 (Goncalves et al., 2016), and Rab1, which (Cooper et al., 2006) promote the clearance of αSyn inclusions and attenuate αSyn-induced toxicity. Furthermore, Rab11a and Rab13 expression enhanced the endocytic recycling and secretion in cells accumulating αSyn inclusions (Goncalves et al., 2016). In contrast, Rab11 regulates the recycling of extracellular αSyn (Liu et al., 2009) and modulates αSyn-mediated defects in synaptic transmission and locomotor behavior in experimental PD models (Breda et al., 2015). This is particularly interesting as Rab11 has been identified as a major regulator of endosomal recycling (Grant and Donaldson, 2009) and controls the secretion of smaller αSyn oligomers by exosomes (Poehler et al., 2014).

Exosomes are nano-sized vesicles (50–150 nm) that are released from cells into the extracellular space (Thery et al., 2002). Exosomes circulate throughout the body and readily cross the blood–brain and other barriers. Great interest in exosomes is emerging because of their potential role in disease progression as well as their possible use in early biomarker discovery (Sarko and McKinney, 2017) and drug delivery (Luan et al., 2017). Toxicology researchers are building upon the discovery that environmental toxicants change the exosome signature of human health conditions such as cancer and neurodegenerative diseases (Harischandra et al., 2017; Munson et al., 2018; Ngalame et al., 2018). In this regard, the impact that Mn exposure has on the neuronal exosome signature and its subsequent effect on neuroinflammation and neurodegeneration have been studied in great detail in our laboratory (Harischandra et al., 2015a, b, 2017, 2018, 2019). We have shown that Mn exposure significantly upregulates the small GTPase Rab27a, which mediates the membrane fusion of multivesicular bodies (Pfeffer, 2010) that subsequently release exosomes into the extracellular environment (Harischandra et al., 2018). Furthermore, our miRNA profiling analysis of Mn-induced neuronal exosomes indicates increased expression of certain miRNAs (e.g., miR-210, miR-325, miR-125b, miR-450b) known to control key biological mechanisms, including inflammation, autophagy, protein aggregation, and hypoxia (Harischandra et al., 2018). In subsequent studies, we show how Mn exposure promotes the exosomal secretion of aggregated αSyn into the extracellular medium. These exosomes were endocytosed through caveolae-mediated endocytosis, thereby inducing neuroinflammation that subsequently evoked neurodegenerative processes in both cell culture and animal models (Harischandra et al., 2019). Interestingly, serum exosome samples collected from welders chronically exposed to Mn-containing welding fumes show increased misfolded αSyn in their exosomes, further implicating environmental Mn exposure in developing Parkinsonism (Harischandra et al., 2019). In parallel studies, we revealed Mn’s role in inflammasome activation in microglial cells. We found that Mn acts as signal 2 for NLRP3 inflammasome activation in LPS-primed microglial cells, triggering the exosomal release of ACS “prionoids,” resulting in inflammasome propagation (Sarkar et al., 2019). Together, our results highlight Mn’s role in modulating endosomal trafficking through the exosomal release of cargo capable of triggering neuroinflammation and progressive neurodegeneration.

## Manganese and Neuroinflammation

In addition to the importance of oxidative stress in the Mn-induced dysfunction of dopaminergic neurons, glial cell activation also plays an important role in potentiating Mn neurotoxicity by inducing the release of non-neuronal-derived ROS and inflammatory mediators such as proinflammatory cytokines. The state of glial activation is defined by its morphology and by the proliferation, migration and expression of immune modulatory molecules. The two major types of glial cells in the CNS are astrocytes and microglia, with the latter constituting about 10% of all glial cells in the CNS.

It is now well documented that glial activation is prominent in the brains of humans exposed to Mn, as well as in non-human primate and rodent models of Mn neurotoxicity (Erikson and Aschner, 2006; Huang, 2007; Perl and Olanow, 2007; Cordova et al., 2013). Neuroinflammation is regarded as a key mediator in mechanisms underlying the loss of dopaminergic neurons in PD. The activation of microglia plays a major role in the response to environmental stress and immunological challenges by scavenging excess neurotoxins, removing dying cells and cellular debris, and releasing proinflammatory cytokines (Carson et al., 2007; Tansey et al., 2008). Inducible nitric oxide synthase (iNOS), which produces large amounts of nitric oxide (NO), is released by microglia in response to inflammatory mediators such as LPS and cytokines. The levels of NO are reported to be elevated in the CNS of human PD cases and in animal models of PD (Mogi et al., 1994). Consistent with this finding, iNOS KO animals are resistant to MPTP-induced dopaminergic neuronal loss in the SN (Przedborski and Vila, 2003). The transcription factor NF-κB, required for transcribing proinflammatory molecules, is also activated in the SN of PD patients and MPTP-treated mice (Ghosh et al., 2007). In contrast to microglia, astrocytes do not attack any pathological targets, but instead produce factors that mediate inflammatory reactions seen in the SN of PD brains (Miklossy et al., 2006). Activated astroglial cells were recently found in human PD brains and in the MPTP mouse model of PD (Ghosh et al., 2007; Ghosh et al., 2009).

Astrocytes play a major role in Mn-induced neuroinflammation as they represent a “hub” for brain Mn homeostasis (Wedler and Denman, 1984). The transferrin receptors found on astrocytes readily bind to Tf-Mn^3+^, so it is not surprising to find more Mn in astrocytes than in any other neural cell types. Indeed, astrocytes can exhibit Mn concentrations 10- to 50-fold greater than those measured in neurons, making them more susceptible to Mn toxicity than other cell types. During glutamate-induced excitotoxicity, excess glutamate abruptly increases intracellular Ca^2+^ to levels that block Mn^2+^ uptake, prompting a release of mitochondrial Mn^2+^ into the cytosol. High levels of cytosolic Mn^2+^ in astrocytes activate glutamine synthetase, which removes excess glutamate (Wedler et al., 1994). However, excessive extracellular Mn^2+^ can disrupt intracellular Ca^2+^ signaling in astrocytes by competitively occupying Ca^2+^-binding sites, thus interfering with mitochondrial Ca^2+^ homeostasis (Farina et al., 2013), which triggers astrogliosis. In addition, Mn^3+^ causes astrocyte swelling via oxidative/nitrosative pathways (Rama Rao et al., 2007). Increased Mn levels in astrocytes elevate the expression of proinflammatory signals such as iNOS and IL-6 (Moreno et al., 2008). *In vitro* studies show that Mn-treated astrocytes use larger amounts of L-arginine, which is a substrate for NO (Hazell and Norenberg, 1998). While timely expression of these signals is necessary in response to neuronal stress or cellular damage, excessive production is counter-productive, often exacerbating the toxic insult. Microarray gene expression profiling of primary human astrocytes exposed to Mn reveals an upregulation of genes encoding proinflammatory cytokines with a concurrent downregulation of genes involved in cell cycle regulation and DNA replication and repair (Sengupta et al., 2007).

The glutamate-GABA cycle (GGC) is important especially in the context of astrocyte-neuron metabolism. The AA glutamine is a precursor for the production of both glutamate and GABA (Bak et al., 2006). Deamidation of neuronal glutamine to glutamate produces ammonia, which is then transferred to astrocytes and utilized in the amidation of glutamate. Glutamine released by astrocytes is taken up by glutamatergic and GABAergic neurons that incidentally show projections in the basal ganglia and help regulate voluntary movements (Sidoryk-Wegrzynowicz and Aschner, 2013). However, in response to excessive Mn in the brain, Mn rapidly enters astrocytic mitochondria. As mentioned in the previous section, high levels of mitochondrial Mn impair cellular respiration and prevent the production and activation of glutathione peroxidase (GPx). Taken together, astrocytes appear to be particularly affected by a disruption of Mn homeostasis in the brain. This in turn could negatively affect GABAergic and glutamatergic projections in the basal ganglia, leading to the motor deficits characterizing Mn neurotoxicity.

## Manganese in Oxidative Stress and Neurodegeneration

Although the mechanisms of Mn-induced nigrostriatal cell death are not well characterized, Mn neurotoxicity appears to be regulated by multiple factors, including oxidative injury, mitochondrial dysfunction, protein misfolding, and neuroinflammation.

**FIGURE 4:**
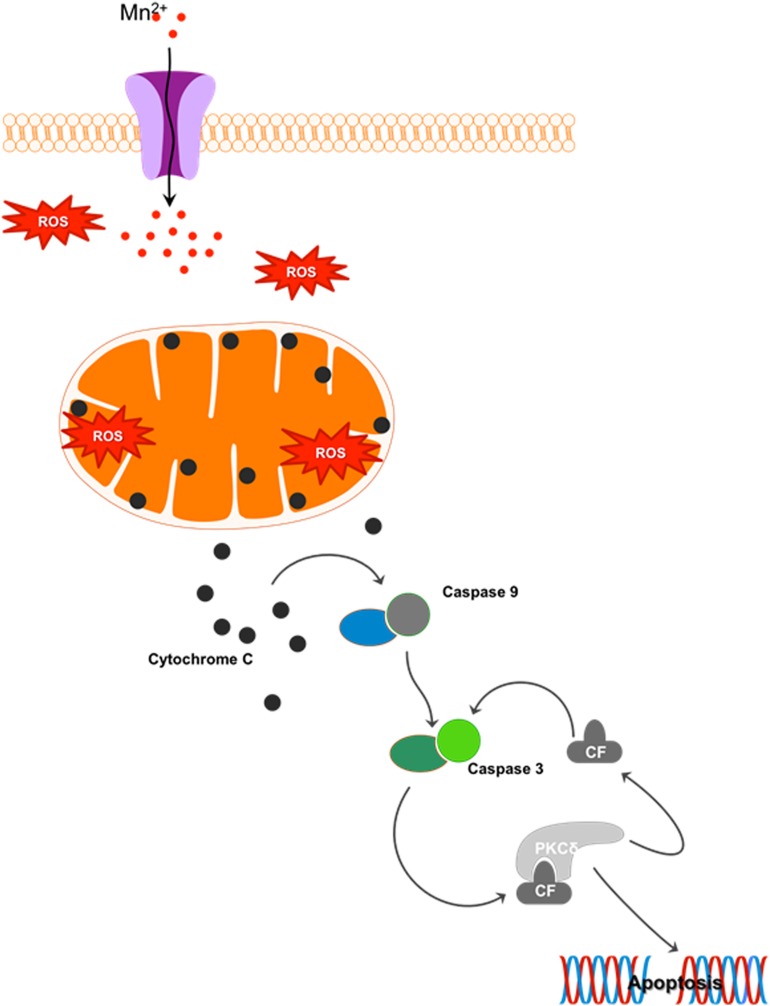
Mn-induced cell death: Cellular Mn homeostasis is dependent upon efficient uptake, retention and excretion by various cell receptors and/or ion channels. In the presence of excess Mn, homeostatic mechanisms will down-regulate the receptors involved in the uptake of this metal, while up-regulating those involved in its release from the cell. However, during chronic exposure to high concentrations of Mn, these system checks are not maintained. Continued uptake of Mn under these conditions increases the production of reactive oxygen species (ROS), leading to mitochondrial dysfunction. Impaired mitochondrial function leads to the release of cytochrome c, activating the apoptosis initiator caspase-9, which in turn cleaves caspase-3. The cleaved fragment of caspase-3 interacts with protein kinase C delta (PKCδ), a pro-apoptotic protein. Caspase-3-mediated proteolytic cleavage of PKCδ leads to DNA fragmentation and apoptosis.

Mn is a redox-active metal whose high reduction potential aids the removal of harmful byproducts of oxygen metabolism, such as superoxide (O_2_^.–^) and hydrogen peroxide (H_2_O_2_), when as a cofactor it forms manganese superoxide dismutase (MnSOD). However, when allowed to accumulate, Mn exacerbates oxidative damage. At just 2% of body weight while consuming 20% of the total oxygen and calories, the brain is highly metabolically active and hence highly susceptible to oxidative damage. Since Mn is known to accumulate in the globus pallidus and striatum, these regions are especially vulnerable to oxidative injury because of their intense oxygen consumption, significant dopamine content, and their high content of non-heme iron. A recent study evaluating the effect of Mn on dopamine transporter (DAT)-transfected and non-transfected HEK cells shows that Mn prevents dopamine reuptake in transfected cells and also mobilizes DAT receptors from the cell surface to intracellular compartments. Consequently, dopamine-induced cell toxicity is observed (Roth et al., 2013). Our laboratory systematically characterized the cell signaling mechanisms underlying Mn-induced oxidative stress. We showed that Mn treatment in rat-derived mesencephalic dopaminergic neuronal (N27) cells increases reactive oxygen species (ROS) production (Harischandra et al., 2015a) that can sequentially activate proapoptotic processes like mitochondrial cytochrome *c* release, caspase-3 activation, and DNA fragmentation. This mitochondria-dependent apoptotic cascade did not involve caspase-8 activation, but was triggered by the Mn treatment (Figure 4) (Latchoumycandane et al., 2005). Moreover, the redox-sensitive protein kinase C delta (PKCδ), involved in neurodegenerative disorders such as AD, prion disease, and PD (Kanthasamy et al., 2006; Ciccocioppo et al., 2008; Jin et al., 2011; Harischandra et al., 2014), is reported to be a key mediator in Mn-induced apoptosis (Anantharam et al., 2002; Latchoumycandane et al., 2005). Later studies in differentiated N27 cells also demonstrate that chronic low-dose Mn exposure impairs tyrosine hydroxylase (TH), the rate-limiting enzyme in dopamine synthesis, through activation of PKCδ and protein phosphatase-2A (PP2A) activity (Zhang et al., 2011). Notably, *in vitro* and *in vivo* administration of the hydrophilic antioxidant vitamin E analog trolox (6-hydroxy-2,5,7,8-tetramethylchroman-2-carboxylic acid) reverses Mn-induced neurotoxicity and rescues dysfunctional dopaminergic transmission and Mn-induced motor coordination deficits (Milatovic et al., 2011; Cordova et al., 2013), further emphasizing the relationship between oxidative stress and Mn-related neurodegeneration.

The neurotransmitter dopamine belongs to the catecholamine and phenethylamine families. The chemical structure of catecholamines predisposes them to oxidation, and their well-characterized metabolic routes can yield quinones and free radicals, suggesting that dopamine may also serve as a neurotoxin contributing to the neurodegenerative process through oxidative metabolism. By promoting dopamine auto-oxidation, Mn potentiates dopamine toxicity in high Mn-accumulating areas of the brain (e.g., globus pallidus and striatum). Under homeostatic conditions, monoamine oxidases (MAO) enzymatically oxidize dopamine to produce dihydroxyphenylacetic acid (DOPAC), which catechol-*O*-methyltransferase (COMT) methylates to homovanillic acid (HVA). Alternatively, COMT can convert dopamine to 3-methoxytyramine (3-MT), which MAO then oxidizes to HVA. H_2_O_2_ is another byproduct of this dopamine turnover or deamination, generating inherent oxidative stress conditions in the nigrostriatal system. Dopamine can also be non-enzymatically oxidized by molecular oxygen, yielding H_2_O_2_ and quinones. These quinones also undergo intramolecular cyclization and oxidative reactions to produce neuromelanin (Graham, 1978; Hermida-Ameijeiras et al., 2004). In dopaminergic SN neurons, neuromelanin augments dopamine’s vulnerability to auto-oxidation through quinone modification (Graham, 1978). Therefore, the degradation of dopamine, either enzymatically or non-enzymatically, produces H_2_O_2_. Two prominent Mn valence states, Mn^2+^ and Mn^3+^, are found in biological systems. In the presence of high levels of divalent Mn^2+^, H_2_O_2_ can convert to highly toxic hydroxyl radicals (${}^{\cdot }$OH) via the Fenton reaction. But because of its higher oxidative state, Ali et al. (1995) found Mn^3+^ to be an order of magnitude more cytotoxic than Mn^2+^ in Mn-dosed rats. In fact, Mn^3+^-induced dopamine oxidation, generating quinones and H_2_O_2_, appears to be independent of oxygen and far more rapid than that mediated by Mn^2+^ (Archibald and Tyree, 1987). Since Mn^2+^ can readily oxidize to Mn^3+^ in the human brain via superoxides, the auto-oxidation of catecholamines can only further potentiate oxidative stress.

Impairment of the cellular antioxidant machinery, causing an imbalance between ROS generation and its elimination, plays a major role in the development of certain neurodegenerative processes. The antioxidant glutathione (GSH), present in both neurons and astrocytes, provides the first line of cellular defense against ROS. GSH actively disposes of exogenous peroxides by acting as a co-substrate in reactions catalyzed by GPx, thus playing important functional roles in the CNS. Altered striatal concentrations of GSH, glutathione disulfide (GSSG), ascorbic acid, malondialdehyde (MDA), and the activities of glutathione reductase (GR) and GPx have been previously reported with Mn neurotoxicity, suggesting that an impaired neuronal antioxidant system renders the brain susceptible to Mn-induced neurotoxicity (Chen and Liao, 2002; Dukhande et al., 2006; Maddirala et al., 2015). Moreover, inhibiting GSH synthesis potentiates the Mn-induced increase in inosine, hypoxanthine, xanthine, and uric acid levels in the striatum and brainstem of aged rats (Desole et al., 2000), indicating that Mn-induced cytotoxicity is mediated through mitochondrial dysfunction. Therefore, the specific vulnerability of dopamine neurons to Mn plays a pivotal role in impairing cellular antioxidant defenses, wherein breakdown of the mitochondrial oxidative energy metabolism cascade leads to dopaminergic cell death. Excess ROS fuels the oxidation of membrane polyunsaturated fatty acids (PUFA), yielding numerous arachidonic acid (ARA) peroxidation products, including reactive aldehydes such as 4-hydroxy-*trans*-2-nonenal (4-HNE), 4-oxo-*trans*-2-nonenal (4-ONE), MDA, acrolein, F_2_-isoprostanes (F_2_-IsoPs), and isofurans (Esterbauer et al., 1991; Aluru et al., 2015). The lipid ARA had been released from neural membrane glycerophospholipids through the activation of cytosolic phospholipases A_2_ (cPLA_2_), which are enzymes coupled to NMDA receptors (Farooqui and Horrocks, 2007; Farooqui and Farooqui, 2011). Since most biological membranes of cells and organelles are composed of PUFA, lipid peroxidation is the main molecular mechanism involved in the oxidative damage to cell structures and in toxicity-mediated cell death. Consistent with these observations, primary rat cortical neurons exposed to a very high Mn dose (500 μM) for 6 h show structural damage to neurons and a roughly 50% increase in F_2_-IsoPs levels compared to controls (Milatovic and Aschner, 2009). Likewise, in primary astrocyte cultures exposed to the same experimental conditions, F_2_-IsoPs levels increased 51% compared to control cultures (Milatovic et al., 2007). However, the direct role of Mn in CNS toxicity associated with lipid peroxidation remains debatable as some investigators argue that *in vivo* administration of Mn alters cellular Ferrous (Fe^2+^), which plays a permissive role in increasing lipid peroxidation and augmenting neuronal vulnerability (Shukla and Chandra, 1981; Chen et al., 2000, 2006).

Moreover, dopamine-derived quinones are known to bind and modify several PD-related proteins such as αSyn, DJ-1, and parkin (Conway et al., 2001; LaVoie et al., 2005; Girotto et al., 2012). However, of all the cellular macromolecules prone to oxidative damage, damaged nucleic acids are particularly hazardous due to the elevated risk of potentially irreparable genetic base mutations. Among the five nucleobases, guanine is the most susceptible to hydroxyl radical-mediated oxidation (Cooke et al., 2003; Cerchiaro et al., 2009), which produces the well-studied oxidized DNA product 8-hydroxyguanosine (8-OHG). Interestingly, elevated 8-OHG as well as reduced 8-hydroxyl-2-deoxyguanosine (8-OHdG) have been observed in the SN and cerebrospinal fluid (CSF) of PD patients (Zhang et al., 1999; Isobe et al., 2010). In contrast, *in vitro* studies of Mn toxicity reported increased 8-oxo-7,8-dihydro-2′-deoxyguanosine (8-oxodG) content in the DNA of dopamine-treated PC12 cells (Oikawa et al., 2006). Stephenson et al. (2013) have also shown that Mn catalyzes the auto-oxidation of catecholamines in SH-SY5Y cells with the ensuing oxidative damage to thymine and guanine DNA bases, further indicating the damaging effect of Mn-induced semi-quinone radical ions and ROS production on DNA.

Mn preferentially accumulates in mitochondria, through the mitochondrial Ca^2+^ uniporter, where it is mainly bound to mitochondrial membrane or matrix proteins (Gavin et al., 1999). Succinate, malate, and glutamate are important substrates for mitochondrial respiration, but at high concentrations, Mn^2+^ binds to these substrates effectively inhibiting mitochondrial respiration (Gavin et al., 1999). Interference in oxidative phosphorylation triggers the downstream release of inflammatory signals, leading ultimately to apoptosis. Recent evidence sheds light on Mn-induced ER stress and ER-mediated cellular apoptosis. Rats given three different doses of Mn for 4 weeks showed a dose-dependent increase in apoptotic cells in the striatum, as evidenced by chromatin condensation, as well as up-regulation of markers of mitochondrial and ER stress-mediated apoptosis (Wang et al., 2015). Furthermore, Mn induces the transcriptional and translational up-regulation of αSyn (Li et al., 2010), promoting susceptibility to Mn-induced neurotoxicity through ERK1/2 MAPK activation, NF-κB nuclear translocation, and activation of apoptotic signaling cascades leading to dopaminergic cell death (Li et al., 2010; Prabhakaran et al., 2011).

Mn affects not only cellular viability, but also various factors involved in neurotransmitter regulation. Acetylcholine esterase (AChE) is an enzyme that hydrolyses acetylcholine (ACh), thus regulating its availability in the synaptic cleft (Whittaker, 1990; Pohanka, 2012). Chronic exposure to high levels of Mn can inhibit the activity of AChE, thereby allowing ACh to accumulate in the synaptic cleft and subsequently overstimulating muscarinic and nicotinic ACh receptors. While the precise mechanism has not been determined, inhibiting AChE increases levels of ROS and RNS (Milatovic et al., 2006; Santos et al., 2012), which further leads to lipid peroxidation as well as production of citrulline, a marker of RNS activity. Ali et al. (1983) reported that Mn overexposure in rats on a low-protein diet reduces the level of GABA in the brain while increasing the animals’ susceptibility to seizures. However, the effect depended on the treatment regime and age of rats. For instance, low-dose Mn given thrice weekly for 5 weeks increased GABA levels (Takagi et al., 1990). Additional mechanistic studies are needed to better understand Mn’s role in GABA dysregulation. In the case of glutamate, high levels of Mn in the brain may trigger constitutive NMDA activation leading to excitotoxic-related neuronal death. Once released into the synaptic cleft, most glutamate is removed by astrocytes via the glutamate-aspartate transporter (GLAST). However, high levels of extracellular Mn^2+^ decrease the expression of GLAST and induce astrocyte apoptosis (Erikson et al., 2002). Chronic exposure to Mn can also increase the amplitude of excitatory postsynaptic potentials (EPSPs) in striatal neurons. With respect to the neurotransmitter dopamine, Ingersoll et al. (1999) demonstrated Mn transport to dopaminergic neurons via DAT. Another study done on DAT^–/–^ mice receiving high doses of Mn reported a lower amount of striatal Mn compared to WT mice given the same dose. Interestingly, only the normally DAT-rich region of the striatum showed this contrasting pattern, which was not seen in areas not expressing DAT (Erikson et al., 2005). Young non-human primates exposed to a low dose of Mn twice weekly for about 9 weeks show retracted microglial processes even while dopaminergic neurons remained unchanged (Verina et al., 2011). More information is needed on the effect of this microglial disturbance on nigrostriatal neurons.

To conclude, Mn influx and efflux are tightly controlled in the body by various receptors and ion channels. However, overexposure to Mn can lead to the toxic accumulation of Mn in the brain, especially in the basal ganglia, causing hyperactivity of cortico-striatal neurons. While contradictory evidence arises from different dose regimens, in general Mn also impairs the regulation of neurotransmitters, such as dopamine, glutamate, and GABA by inhibiting the enzyme activity that regulates optimum neurotransmitter levels. High levels of glutamate and/or acetylcholine in the synaptic cleft overstimulate NMDA receptors leading to excitotoxic neuronal death. Mn may get transported into dopaminergic neurons via DAT. Excess cellular Mn^2+^ disrupts Ca^2+^ homeostasis in cells, leading to decreased dopamine production and neuronal death. Mn also causes ER and mitochondrial stress leading to neuronal apoptosis and/or gliosis. In light of the mounting evidence pointing to the deleterious effects of Mn on neurons and glia, researchers are examining the use of metal chelators and antioxidants as therapeutic interventions against manganism.

## Manganese in Other Diseases

Until the last decade, Mn neurotoxicity was mainly associated with Parkinsonism, and very little attention had been given to its potential role in other neurodegenerative diseases. However, with growing interest in the neurobiology of heavy metals, Mn has now been linked to other major neurodegenerative diseases such as HD and prion diseases (Choi et al., 2010; Martin et al., 2011; Kumar et al., 2015). Furthermore, gene expression in the frontal cortex of *cynomolgus macaques* exposed to various Mn doses indicates that the amyloid-β (Aβ) precursor-like protein 1 (APLP1) of the amyloid precursor family was highly up-regulated, thereby linking Mn exposure to AD (Guilarte et al., 2008). Along with this gene array analysis, immunochemistry revealed the presence of Aβ plaques and αSyn aggregates, which have been linked to PD as well as AD, and which have also been seen in the gray and white matter of Mn-exposed animals (Guilarte, 2010).

In contrast to Mn-induced Parkinsonism, the pathogenesis of HD, an autosomal dominant disorder characterized by the neurodegeneration of medium spiny neurons in the striatum, appears to involve a Mn transport deficiency (Kumar et al., 2015). Recent experiments carried out with immortalized mutant HD cell lines (SThdh^Q7/Q7^ and SThdh^Q111/Q111^) show reduced TfR levels and substantial deficits in Mn uptake (Williams et al., 2010). In follow-up studies, YAC128 HD transgenic mice accumulated less Mn in their basal ganglia, including their striata, which are focal regions for both HD neuropathology and Mn accumulation (Madison et al., 2012). Furthermore, transition metal analysis of HD patients has shown significantly increased iron together with significantly decreased cortical copper and Mn (Rosas et al., 2012), further supporting the role of Mn in HD.

Prion protein (PrP) is widely known for its association with transmissible spongiform encephalopathies (TSE), a class of neurodegenerative diseases caused by the accumulation of an abnormal isoform of the prion protein known as PrP^Sc^ (Jin et al., 2015a). The cellular prion protein PrP^C^ has a high-binding affinity for divalent metals. In fact, above-normal Mn content has been detected in the blood and brains of humans infected with Creutzfeldt–Jakob disease (CJD), in scrapie-infected mice, and in bovines infected with bovine spongiform encephalopathy (BSE) (Wong et al., 2001; Hesketh et al., 2007; Hesketh et al., 2008). The binding of Mn to prion protein mitigates Mn’s neurotoxicity during the early acute phase of Mn exposure (Choi et al., 2007). However, prolonged Mn exposure alters the stability of prion proteins without changing gene transcription (Choi et al., 2010), suggesting that Mn contributes to prion protein misfolding and prion disease pathogenesis. Interestingly, prion proteins survive significantly longer in a Mn-enriched soil matrix (Davies and Brown, 2009), a finding with important implications for the environmental transmissibility of PrP^Sc^. The role of Mn in TSE was further validated by our lab’s discovery that it enhances the ability of the pathogenic PrP^Sc^ isoform to regulate Mn homeostasis (Martin et al., 2011) and by Davies and Brown (2009) reporting that Mn increases the infectivity of scrapie-infected cells. Therefore, deepening our understanding of how metals interact with disease-specific proteins will provide further insight into the pathogenesis and potential treatment of neurodegenerative diseases.

## Future Directions

Our review of existing literature related to Mn overexposure and associated health issues has revealed genetic, sex- and age-related susceptibilities, signaling cascades involved in Mn neurotoxicity, and comparisons between PD and other motor disorders. Yet, many aspects of Mn overexposure and homeostasis remain largely understudied. For example, early childhood exposure to drinking water containing elevated Mn levels has been conclusively shown to compromise certain aspects of memory and learning; however, how absorption of excessive levels of Mn via the GIT leads to cognitive deficits (a CNS component) is still largely unknown. Secondly, given that the nasal tract and GIT are two well-known microbial environments, does overexposure to Mn via inhalation or ingestion alter the community composition of nasal or gut microbes or otherwise cause dysbiosis? Are changes in microbial populations offset by other lines of host defense against Mn toxicity or do these changes exacerbate the neuropathology? Thirdly, MRI and positron emission tomography (PET) on Mn-exposed individuals have shown changes in brain Mn accumulation and dopamine and GABA neurotransmitter levels. Yet despite the strong links between manganism and Parkinsonism, no case control study has examined the extent of elevated Mn accumulation in the brain and its associated neuropathology, including the presence of Lewy bodies/neurites and elevated phospho-α-synuclein expression. Additionally, longitudinal studies examining the immediate, intermediate and long-term effects of elevated Mn exposure on children and adults are needed to identify age- and sex-specific susceptibilities, and potential biological, psychological and cognitive biomarkers. Lastly, studies systematically identifying Mn exposure limits based on age, sex, and exposure duration as well as changes in signaling cascades associated with metal homeostasis and protein aggregation need to be conducted.

## Conclusion

**FIGURE 5:**
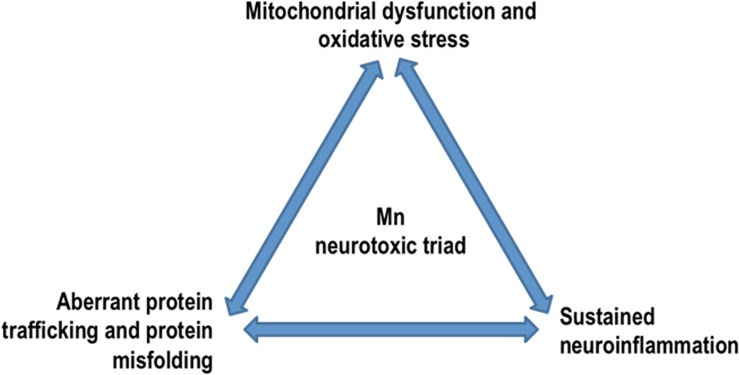
Mn neurotoxic triad: by dysregulating key protein degradative and trafficking pathways, Mn overexposure results in a ‘neurotoxic triad’ comprising mitochondrial dysfunction and oxidative stress, protein misfolding and trafficking, and neuroinflammation.

Chronic exposure to excessive Mn induces various neurological and psychiatric symptoms. While the body can efficiently remove excess Mn, primarily through the gut and liver, the brain cannot. Because of direct passage via the nasal neuroepithelium, inhaling large doses of Mn can lead to its accumulation in the brain’s basal ganglia. Astrocytes are particularly sensitive to Mn toxicity and may compound neuroinflammation by releasing pro-inflammatory cytokines in response to excess Mn. Mn can also bind to substrates of oxidative phosphorylation, thus inhibiting mitochondrial respiration and thereby augmenting oxidative stress. Chronic exposure to Mn causes benign α-synuclein monomers, present in all neurons, to undergo a conformational change to the oligomeric structures that are toxic to neurons. Additionally, Mn dysregulates key protein degradative and trafficking pathways including proteasomes, autophagy, and endosomal trafficking. Taken together, we entertain the notion that a ‘neurotoxic triad,’ comprising mitochondrial dysfunction and oxidative stress, protein misfolding and trafficking, and neuroinflammation, plays a major pathogenic role in Mn neurotoxicity (Figure 5). Beyond its effects on the CNS, excess Mn also interferes with the body’s iron metabolism and can cause kidney failure. Early detection and chelation therapy can effectively reverse the harmful effects caused by this metal; however, if it progresses untreated, it can cause severe neurological and physiological defects. As with any metal, the bioaccumulation and teratogenic effects of Mn remain a risk, yet this aspect has not been studied in detail. Similarly, an in-depth study of Mn’s role in protein misfolding and the upregulation of genetic markers for various neurological diseases in humans must be conducted. By combining the results of epidemiological surveys with human case studies as well as mechanistic studies done in *in vitro* and in animal models of Mn toxicity, we will eventually decipher the causes and symptoms of neurodegeneration caused by Mn toxicity well enough to develop effective therapeutic strategies that can be readily used against environmentally linked PD and related chronic neurodegenerative diseases.

## Author Contributions

DSH and SG conceived and wrote the article. GZ, HJ, AK, VA, and AGK provided intellectual input for review content and edited the manuscript. All authors read and approved the manuscript.

## Conflict of Interest Statement

AGK and VA have an equity interest in PK Biosciences Corporation located in Ames, IA, United States. The terms of this arrangement have been reviewed and approved by ISU in accordance with its conflict of interest policies. The remaining authors declare that the research was conducted in the absence of any commercial or financial relationships that could be construed as a potential conflict of interest.
